# Translational targeting of inflammation and fibrosis in frozen shoulder: Molecular dissection of the T cell/IL-17A axis

**DOI:** 10.1073/pnas.2102715118

**Published:** 2021-09-20

**Authors:** Moeed Akbar, Lindsay A. N. Crowe, Michael McLean, Emma Garcia-Melchor, Lucy MacDonald, Kristyn Carter, Umberto G. Fazzi, David Martin, Angus Arthur, James H. Reilly, Iain B. McInnes, Neal L. Millar

**Affiliations:** ^a^Institute of Infection, Immunity and Inflammation, College of Medical, Veterinary and Life Sciences,University of Glasgow, Glasgow G12 8QQ, United Kingdom;; ^b^Department of Orthopaedic Surgery, Queen Elizabeth University Hospital, Glasgow G51 4TF, United Kingdom

**Keywords:** frozen shoulder, T cell, IL-17A, inflammation, adhesive capsulitis

## Abstract

Frozen shoulder is a common fibroproliferative disease characterized by the insidious onset of pain and restricted range of shoulder movement with a significant socioeconomic impact. The pathophysiological mechanisms responsible for chronic inflammation and matrix remodeling in this prevalent fibrotic disorder remain unclear; however, increasing evidence implicates dysregulated immunobiology. IL-17A is a key cytokine associated with inflammation and tissue remodeling in numerous musculoskeletal diseases, and thus, we sought to determine the role of IL-17A in the immunopathogenesis of frozen shoulder. We demonstrate an immune cell landscape that switches from a predominantly macrophage population in nondiseased tissue to a T cell–rich environment in disease. Furthermore, we observed a subpopulation of IL-17A–producing T cells capable of inducing profibrotic and inflammatory responses in diseased fibroblasts through enhanced expression of the signaling receptor IL-17RA, rendering diseased cells more sensitive to IL-17A. We further established that the effects of IL-17A on diseased fibroblasts was TRAF-6/NF-κB dependent and could be inhibited by treatment with an IKKβ inhibitor or anti–IL-17A antibody. Accordingly, targeting of the IL-17A pathway may provide future therapeutic approaches to the management of this common, debilitating disease.

## Significance

Frozen shoulder (FS) is a classic example of a prevalent debilitating pathological inflammatory fibrosis. Using approaches to dissect the molecular pathways subserving FS, we demonstrate the immune cell landscape in FS shifts from primarily macrophages to T cells in disease. We find T cells from FS tissue are able to secrete the inflammatory cytokine IL-17A, and fibroblasts from FS have greater expression of the receptor IL-17RA. Thus, FS fibroblasts produce greater fibrotic and inflammatory responses following IL-17A stimulation, which can be abrogated following NF-κB pathway inhibition or cytokine blockade using an anti–IL-17A antibody. This single-cell dissection of FS highlights a T cell–driven disease with both small molecule and anti-cytokine attenuation of disease-like features.

Frozen shoulder, also known as adhesive capsulitis, is a very common painful condition of the shoulder that can affect up 10% of people of working age (1). Clinically, it manifests as progressively limited joint movement in all directions associated with pain and loss of function (2). Frozen shoulder is more prevalent in women, mainly affecting those in the fifth and sixth decades of life, and is the third most common cause of musculoskeletal disability in the United States and European Union (3). While often thought of as a self-limiting disease, the majority of patients suffer permanent disability at long-term follow up, with some patients reporting painful symptoms at 6-y postdisease onset (4), costing the US healthcare system in excess of $85 million per annum (5). Numerous rehabilitative, medical, and surgical therapies are available (2); however, current research does not support a clear treatment strategy, and none are derived from an understanding of the molecular mechanisms underpinning disease pathology.

The principal structure affected in this disease is the shoulder capsule, with pathological changes culminating in tissue fibrosis. Inflammation is known to play a crucial role in tissue fibrosis, and various proinflammatory cytokines have been implicated in driving the progression of many fibrotic diseases (6–8). Frozen shoulder capsular tissue has demonstrated inflammatory cell influx (9), dysregulated expression of alarmins (HMGB-1, Tenascin C, IL-33, S100A8, S100A9) (10, 11), inflammatory proteins (IL-1α, IL-1β, IL-6, TGF-β, COX-1, COX-2, TNF-α) (12, 13), matrix regulating (matrix metalloproteinases [MMPs] and tissue inhibitors of metalloproteinases [TIMPs]) (12, 14), and markers of stromal fibroblast activation (PDPN, VCAM, MCAM) (15, 16). Thus, the presence of immune cells, dysregulated inflammatory/matrix interactions, and activated fibroblasts allude to a significant and targetable immune component in the pathogenesis of frozen shoulder.

IL-17A is a cytokine known to mediate inflammation (17), fibrosis (18), and pain signaling (19). It is the founding member of the IL-17 cytokine family and the signature cytokine of the Th17 T-helper cell population, although it is also expressed by γδ T cells, innate lymphoid, and natural killer cells (20, 21) The IL-17 receptor family contains five members (A through E), and IL-17A binds and exerts its downstream effects through an IL-17RA/RC complex (21). Fibroblasts are among the most responsive cells to IL-17A and, following IL-17RA/RC binding, induce mitogen-activated protein kinases, nuclear factor kappa-b (NF-κB), phosphoinositide 3 kinase (PI3K), and C/EBP signaling pathways. The resultant IL-17 “signature” includes, but is not limited to, the promotion of proinflammatory cytokine, chemokine, and matrix metalloproteinase expression from multiple target cells, including stromal fibroblasts (17, 18, 22–26). IL-17A signaling has been identified and successfully targeted as a clinical therapy in a host of chronic inflammatory conditions including ankylosing spondylitis and psoriatic arthritis (27–29). Furthermore, we have recently shown the translational potential of IL-17A in the soft tissue musculoskeletal condition, tendinopathy (26, 30).

Frozen shoulder has a cellular and molecular profile that is intriguingly similar to the manifestation of IL-17A signaling. Based on these observations, we hypothesized that IL-17A signaling may be involved in mediating the inflammatory and tissue remodeling changes present in frozen shoulder in a similar manner to that which is involved in tendinopathy (26). The purpose of this study was, firstly, to characterize the immune cell and expression of IL-17A in human frozen shoulder capsule tissue compared to healthy control capsule and, secondly, to interrogate IL-17A signaling in frozen shoulder fibroblasts in vitro to ultimately determine if any pathogenic effects could be impeded. Herein, we report T cell–driven IL-17A production and signaling, which induces the fibrotic features of frozen shoulder. Importantly, we demonstrate that manipulation of cytokine (IL-17A) signaling and response pathways can be a therapeutic opportunity for this debilitating disease.

## Results

### IL-17A–Secreting T Cells in Frozen Shoulder.

**Fig. 1.**
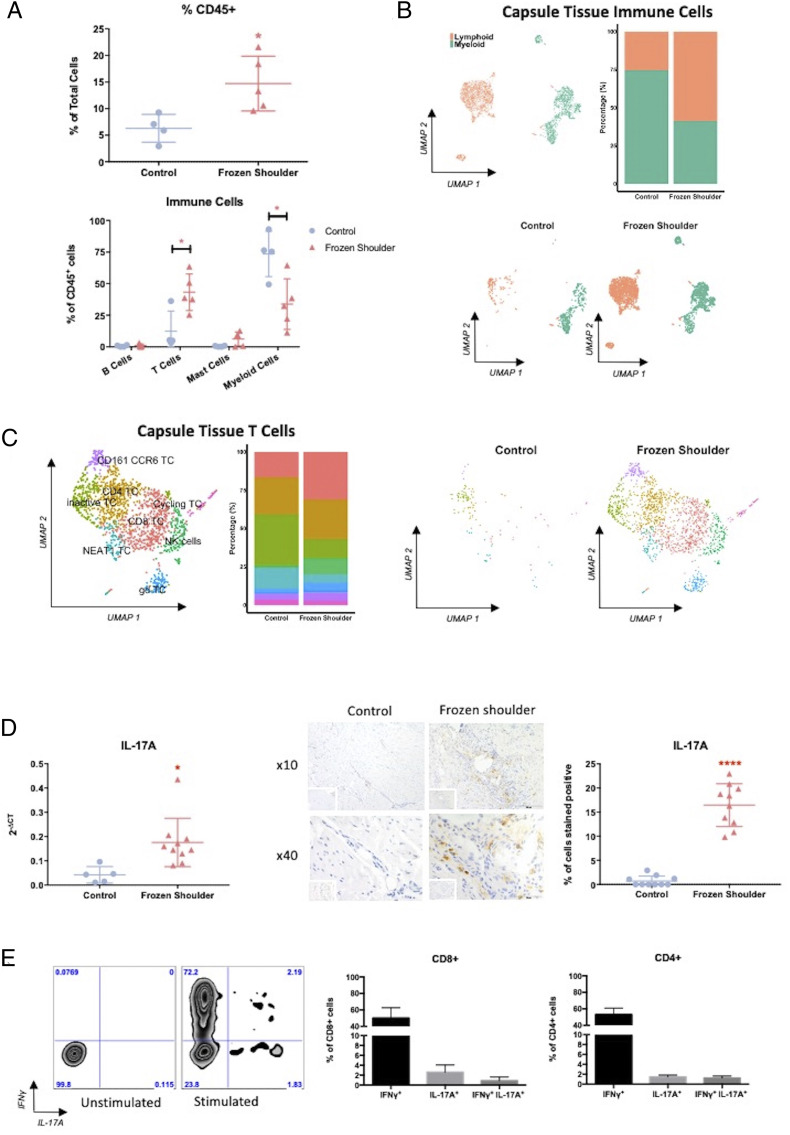
T cells produce IL-17A in frozen shoulder. (*A*) Percentage and subsets of immune cells phenotyped from disaggregated shoulder capsule tissue. Results are mean ± SD, *n* = 4 control capsule and *n* = 5 frozen shoulder. Statistical analysis using unpaired Student’s *t* test. * indicates significant difference from control capsule tissue, *P < 0.05. (*B*) Uniform Manifold Approximation and Projection (UMAP) embedding of single-cell RNA sequencing and distribution of immune cells from all shoulder capsule tissue (*n* = 7, k = 3,347) and split into shoulder capsule of control (*n* = 3, k = 555 and frozen shoulder tissue (*n* = 4, k = 2,792). (*C*) UMAP embedding and distribution of eight delineated T cell populations (CD161 CCR6 T cells, inactive T cells, CD4 T cells, CD8 T cells, natural killer [NK] cells, NEAT1 T Cells, γδ T cells) in all shoulder capsule tissue (*n* = 7, k = 1,562) and split into shoulder capsule of control (*n* = 3, k = 120) and frozen shoulder tissue (*n* = 4, k = 1,442). (*D*) IL-17A gene and protein expression in shoulder capsule tissue. IL-17A expression in control and frozen shoulder capsule, 2^-ΔCT^ relative to GAPDH, mean ± SD, *n* = 5 control capsule and *n* = 10 frozen shoulder. Statistical analysis using unpaired Student’s *t* test, * indicates significant difference from control capsule tissue, *P < 0.05. Shoulder capsule tissue stained for IL-17A, isotype IgG in bottom left corner, using rabbit polyclonal IL-17A antibody at 10× and 40× magnification. Graph illustrates percentage of cells stained positive for IL-17A, mean ± SEM, *n* = 10 for control, *n* = 10 for frozen shoulder tissue. Statistical analysis using unpaired Student’s *t* test, ****P < 0.0001. (*E*) IL-17A is produced by T cells from disaggregated frozen shoulder tissue. Representative flow cytometric plots of unstimulated and stimulated CD3^+^ T cells following intracellular staining with antibodies against IFN-γ and IL-17A. Graph displays the proportion of CD4^+^ and CD8^+^ T cells positive for IFN-γ and IL-17A (*n* = 4).

In order to dissect the role of immune cells in the fibrotic chronicity of frozen shoulder, we initially analyzed the immune cells (CD45^+^) in the shoulder capsule from control and frozen shoulder tissue. Flow cytometry of disaggregated tissue demonstrated increased percentage of CD45^+^ immune cells in frozen shoulder tissue (Fig. 1*A*). Further phenotypic analysis by both flow cytometry and single-cell RNA sequencing showed that while control capsule immune cells were predominantly composed of myeloid cells, diseased tissue from frozen shoulder was mainly comprised of lymphoid cells, primarily T cells (Fig. 1 *A* and *B* and *SI Appendix*, Fig. S3*A*). The myeloid component of immune cells in shoulder capsule tissue can be split into five subpopulations consisting of macrophages, three populations of CD1c dendritic cells (DCs): *CD52*-expressing DCs, IL-1 receptor high DCs, and an inflammatory-type DC population that express cytokines and chemokines such as IL-1β and CXCL8 and CLEC9A DCs (*SI Appendix*, Fig. S3*B*). The myeloid cells found in control capsule tissue are predominantly macrophages that express *LYVE1* and *FOLR2*. However, the myeloid component of immune cells from frozen shoulder tissue contained a greater proportion of DCs, principally *CD52* DCs but with a considerable proportion of *CLEC9A* and inflammatory DCs that are extremely rare in control tissue (*SI Appendix*, Fig. S3*B*). The shift in the myeloid compartment of the shoulder capsule from primarily macrophages in control tissue to DCs in diseased tissue is indicative of a T cell response (31). Further analysis of the T cell constituent from single-cell RNA sequencing data indicated the presence of eight subpopulations of T cells (Fig. 1*C* and *SI Appendix*, Fig. S4*A*). Distinct analysis of the T cell populations present in control tissue demonstrated the primary population to be T cells with an inactive phenotype (32); interestingly, the proportion of this population was reduced in the T cell population present in frozen shoulder tissue (Fig. 1*C* and *SI Appendix*, Fig. S4*A*). This was also the case with *NEAT1*-expressing T cells, which were also less abundant in frozen shoulder tissue compared to control tissue (Fig. 1*C* and *SI Appendix*, Fig. S4*A*). There was a greater percentage of the other six subpopulations of T cells in disease tissue compared to control tissue (Fig. 1*C* and *SI Appendix*, Fig. S4*A*). Of particular note was a CD161 CCR6 population which was expanded in frozen shoulder tissue (Fig.1*C*). This population of cells was positive for *KLRB1*(CD161), *CCR6*, and *RORA* and negative for *CXCR3* (Fig. 1*C* and *SI Appendix*, Fig. S4). This combination of genes has previously been indicative of IL-17A–producing T cells (33, 34). As IL-17A has been shown to be pathogenic in numerous inflammatory diseases (35, 36) as well as a number of fibrotic pathologies (18, 24, 37), we sought to identify whether IL-17A was present in diseased shoulder capsule. Gene expression data demonstrated significantly greater expression of IL-17A in frozen shoulder tissue compared to control (Fig. 1*D*). Furthermore, immunohistochemistry showed the increased presence of IL-17A–positive cells in frozen shoulder tissue around areas of high cellularity (Fig. 1*D*). We subsequently sought to stimulate cells released from disaggregated frozen shoulder tissue ex vivo to identify IL-17A–producing T cells. We found that both CD4^+^ and CD8^+^ T cells from frozen shoulder tissue were able to produce IL-17A following stimulation and intracellular staining (Fig. 1*E*). Together, these data highlight the increased presence of T cells in a chronic fibrotic disorder and their ability to produce IL-17A.

### IL-17A Is Profibrotic in Frozen Shoulder.

**Fig. 2.**
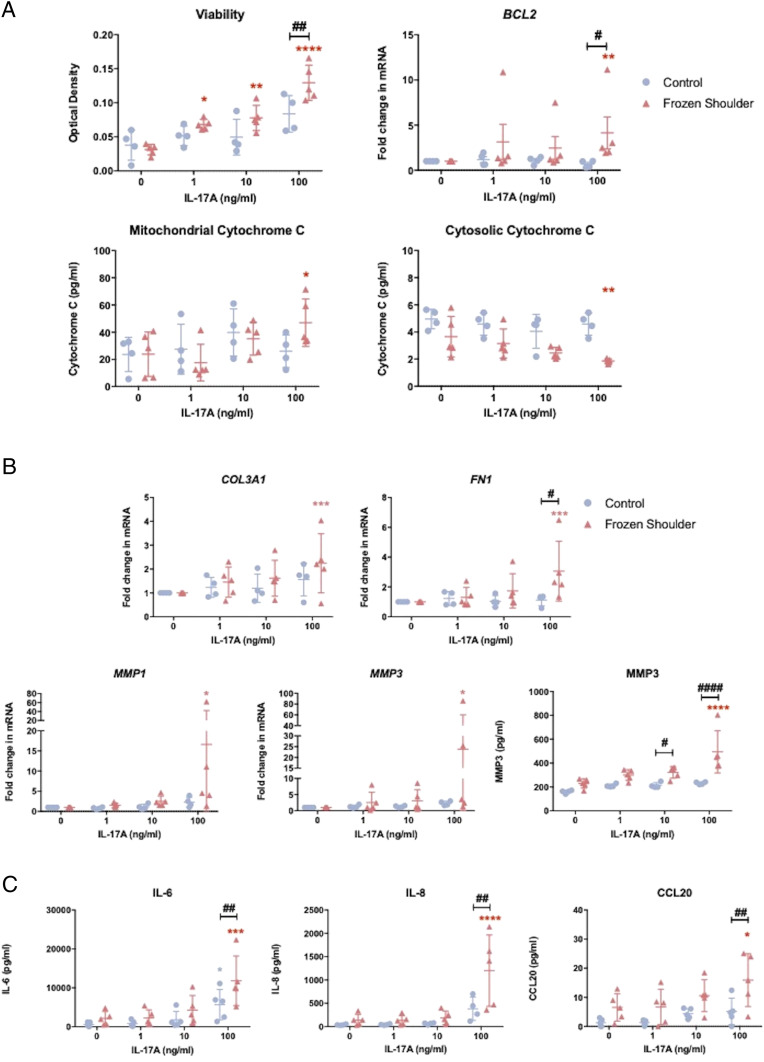
IL-17A induces fibrosis and inflammation in vitro. (*A*) Effect of recombinant IL-17A on control and frozen shoulder fibroblasts viability, *BCL2* gene expression, and mitochondrial and cytosolic cytochrome C content, mean ± SD, *n* = 4 control fibroblasts and *n* = 5 frozen shoulder fibroblasts, * indicates significant difference from untreated cells, *P < 0.05, **P < 0.01, ***P < 0.001, ****P < 0.0001, # indicates significant difference from control fibroblasts, ^#^*P* < 0.05, ^##^*P* < 0.01. (*B*) Effect of IL-17A on *COL3A1*, *FN1*, *MMP1*, *MMP3* gene expression and MMP3 protein secretion. mRNA gene expression expressed as fold change following normalization to housekeeping gene (GAPDH) and then to relevant untreated cells, *n* = 4 control fibroblasts and *n* = 5 frozen shoulder fibroblasts, * indicates significant difference from untreated cells *P < 0.05, **P < 0.01, ***P < 0.001, ****P < 0.0001, # indicates significant difference from control fibroblasts, ^#^*P* < 0.05, ^##^*P* < 0.01 ^###^*P* < 0.001. (*C*) Effect of IL-17A on IL-6, IL-8, CCL20 production from control or frozen shoulder fibroblasts, *n* = 4 control fibroblasts and *n* = 5 frozen shoulder fibroblasts, * indicates significant difference from untreated cells *P < 0.05, **P < 0.01, ***P < 0.001, ****P < 0.0001, # indicates significant difference from control fibroblasts, ^#^*P* < 0.05, ^##^*P* < 0.01 ^###^*P* < 0.001. All statistical analyses use two-way ANOVA with Dunnet’s correction or Sidak’s test for multiple comparisons.

Following confirmation of the increased presence of IL-17A in frozen shoulder, we sought to interrogate the role of IL-17A on the fibrotic features of frozen shoulder in vitro. We were able to demonstrate that IL-17A significantly increased the cell viability of frozen shoulder fibroblasts following IL-17A exposure (Fig. 2*A*). There was also a significant increase in expression of the antiapoptotic gene *BCL2* (38) upon IL-17A exposure (Fig. 2*A*). Additionally, there was also a significant increase in cytochrome C measured in the mitochondria of IL-17A–stimulated frozen shoulder fibroblasts; this was accompanied by a significant decrease in the cytochrome C recorded in the cytosol (Fig. 2*A*). No significant change in cell viability, *BCL2* expression, and mitochondrial or cytosolic cytochrome C was observed in IL-17A–treated control fibroblasts compared to untreated fibroblasts, nor was any significant change detected in cell proliferation and gene expression of proapoptotic (38) *BAX* or cell cycle kinase component *CCND1* (39) in both control and diseased fibroblasts following IL-17A (Fig. 2*A* and *SI Appendix*, Fig. S6*A*). These data suggest IL-17A promotes accumulation of fibroblasts in frozen shoulder capsule as a result of up-regulation of *BCL2*, which inhibits the release of cytochrome C from the mitochondria into the cytosol and subsequently suppresses apoptosis (40, 41) rather than an increase in fibroblast proliferation.

We further explored the fibrotic effects of IL-17A by measuring the gene expression of a number of proteins associated with fibrotic pathology in frozen shoulder (10, 42, 43). IL-17A induced no significant changed in the expression of *COL1A1*, *TNC*, or *α-SMA* in either control or diseased fibroblasts (*SI Appendix*, Fig. S6*B*). However, we did detect significant increase of *COL3A1*, *FN1*, *MMP1*, and *MMP3* gene expression in frozen shoulder fibroblasts following IL-17A exposure (Fig. 2*B*). In addition, we measured significantly greater concentrations of MMP3 in the supernatant of IL-17A–treated frozen shoulder fibroblasts (Fig. 2*B*). No significant increase was observed in the expression of matrix genes or MMP3 in supernatant in control fibroblasts following IL-17A treatment (Fig. 2*B*), indicating the aberrant tissue remodeling effects of IL-17A are selective for diseased cells.

### IL-17A–Induced Inflammation in Frozen Shoulder.

IL-17A is implicated in the pathogenesis of numerous inflammatory diseases (35, 36). Additionally, frozen shoulder has long been recognized as having an inflammatory element. Thus, we investigated the possibility of whether IL-17A could initiate an inflammatory response in frozen shoulder fibroblasts. Following IL-17A treatment of fibroblasts, we assessed the gene expression and protein release of a number of inflammatory mediators. The data demonstrated that IL-17A induced an up-regulation of gene expression and release of IL-6 and IL-8 in frozen shoulder fibroblasts (Fig. 2*C* and *SI Appendix*, Fig. S6*C*). Additionally, the increase in expression of these inflammatory proteins was significantly greater in diseased fibroblasts compared to control fibroblasts (Fig. 2*C* and *SI Appendix*, Fig. S6*C*). We also assessed the gene expression of chemokines associated with T cell recruitment (44–46). There was a significant increase in gene expression of *CCL2*, *CCL5*, and *CCL20* in frozen shoulder fibroblasts; however, no significant increase in the expression of these chemokines was observed in control fibroblasts following IL-17A exposure (Fig. 2*C* and *SI Appendix*, Fig. S6*C*). We also measured the release of CCL20 into the supernatant following IL-17A exposure, as it has previously been shown to be a key recruiter of IL-17A–producing T cells (45). The concentration of CCL20 from supernatant mirrored its gene expression (Fig. 2*C* and *SI Appendix*, Fig. S6*C*) in which a significant increase was measured in the supernatants of diseased fibroblasts exposed to 100 ng/mL; however, no significant increase was measured in the CCL20 release of control fibroblasts exposed to IL-17A. Together, these data indicate diseased fibroblasts from frozen shoulder tissue have greater sensitivity to IL-17A, thereby inducing a greater response upon stimulation in vitro and potentially act to further recruit IL-17A–producing T cells.

### Enhanced IL-17A Signaling in Frozen Shoulder Is NF-κB Dependent.

**Fig. 3.**
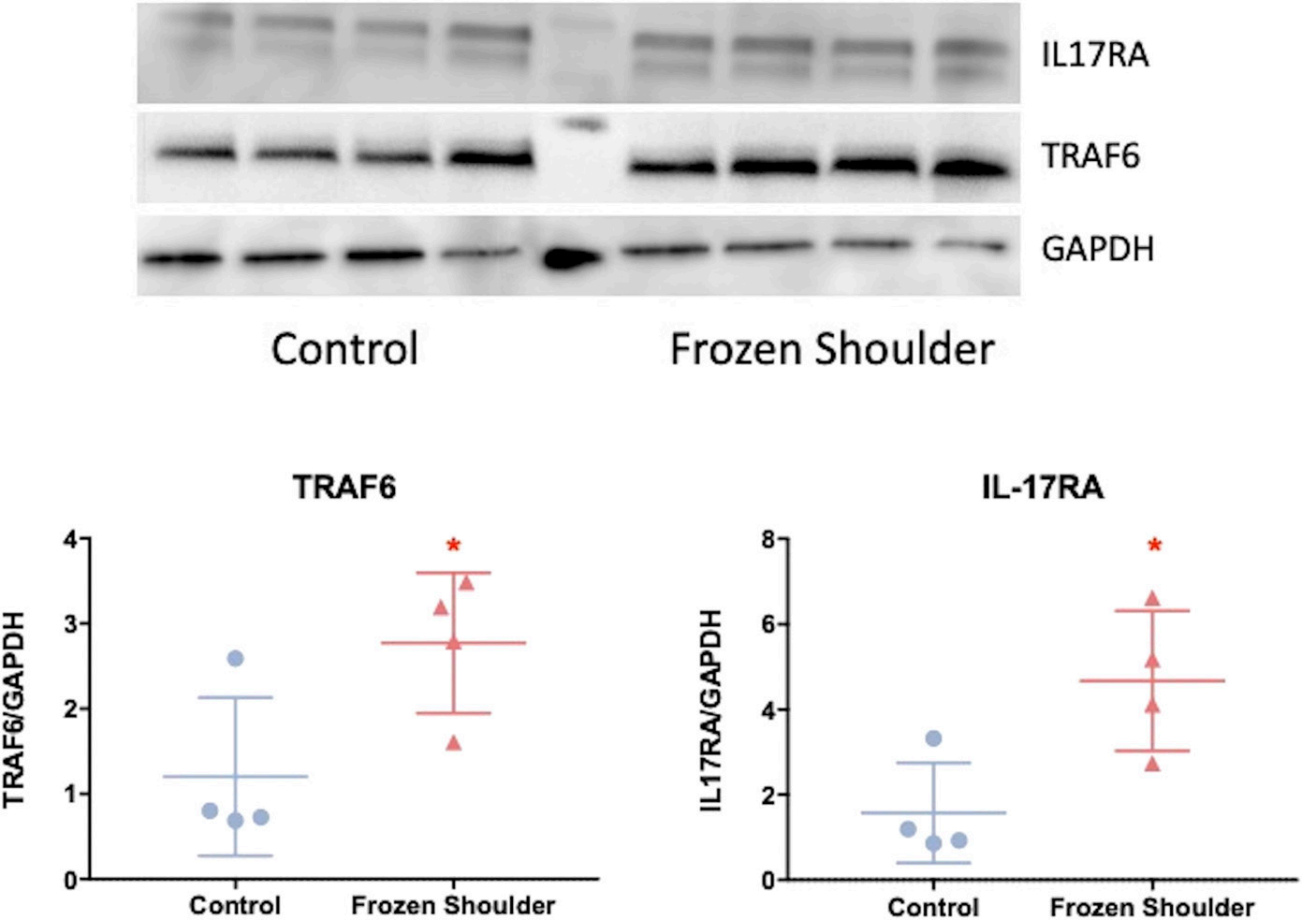
Enhanced IL-17RA expression in frozen shoulder fibroblasts. IL-17RA and TRAF6 protein expression in cultured fibroblasts from control and frozen shoulder capsule. Image of Western blot of protein from control and frozen shoulder fibroblasts immunoblotted for GAPDH, TRAF6, and IL-17RA. Graph illustrates TRAF6 and IL-17RA protein quantification relative to housekeeping (GAPDH), mean ± SD, *n* = 4 control and frozen shoulder fibroblasts. Statistical analysis using Mann–Whitney *U* rank-sum test. * indicates significant difference from control capsule tissue, *P < 0.05.

As IL-17A was found to influence cell survival and matrix and inflammatory proteins in frozen shoulder fibroblasts but not control fibroblasts, we assessed the role of its receptors and associated signaling pathways. Gene expression analysis showed that IL-17RA was the predominant isoform expressed in both control and diseased fibroblasts; however, no significant difference in gene expression of either IL-17 receptor was observed between control and frozen shoulder fibroblasts, nor was there any difference in the protein expression of IL-17RC (*SI Appendix*, Fig. S5 *B* and *C*). However, Fig. 3 demonstrates that diseased fibroblasts show greater protein expression of IL-17RA and its downstream effector TRAF6, supporting the increased sensitivity of diseased cells to IL-17A stimulation compared to control fibroblasts observed in Fig. 2.

**Fig. 4.**
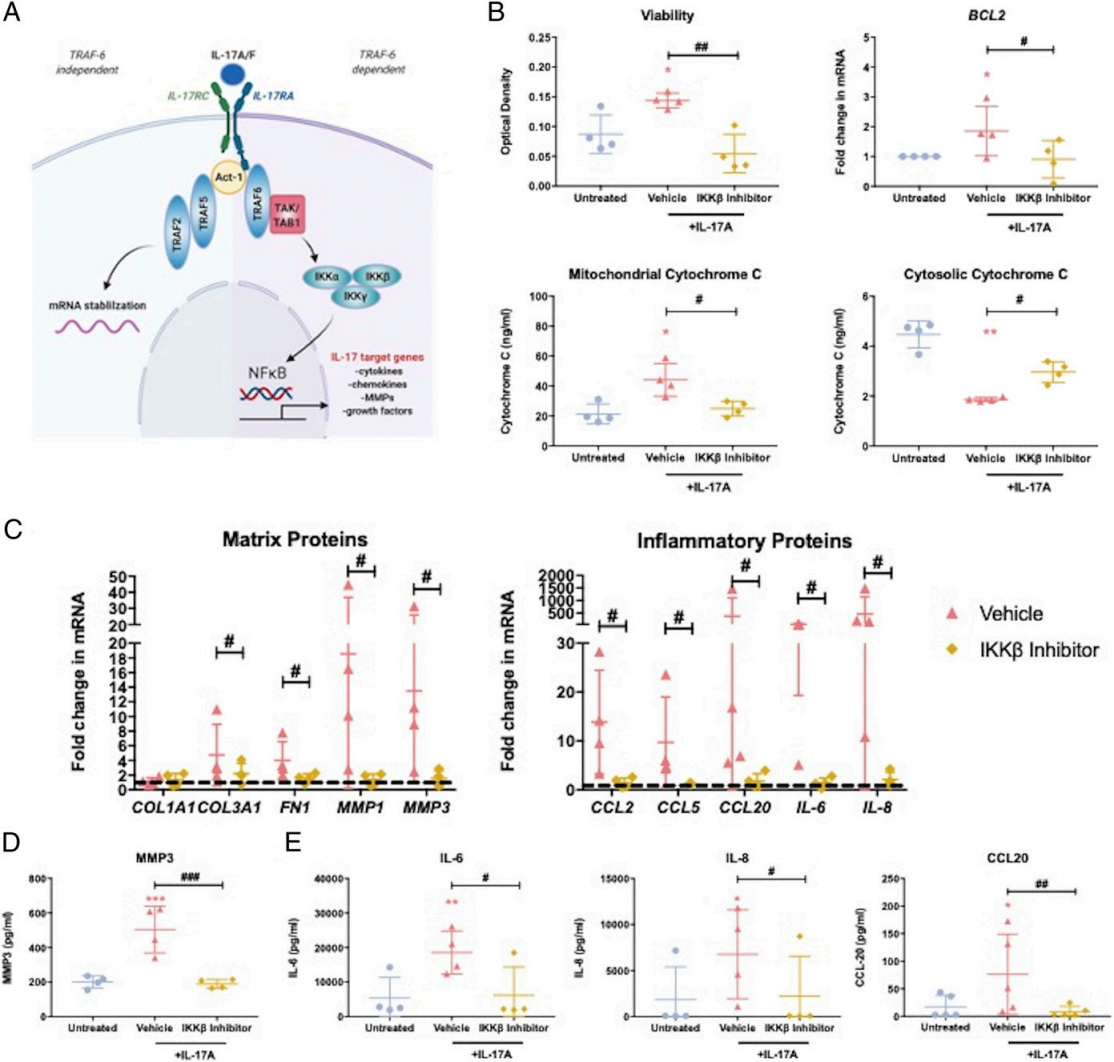
IL-17A signaling is NF-κB dependent in frozen shoulder fibroblasts. (*A*) Schematic diagram of NF-κB pathway following IL-17A stimulation. Frozen shoulder fibroblasts were pretreated with IKKβ inhibitor before exposure to IL-17A (100 ng/mL) (*B*) Fibroblast’s viability, *BCL2* gene expression, and mitochondrial and cytosolic cytochrome C content. (*C*) Gene expression of matrix and inflammatory regulators. mRNA gene expression expressed as fold change following normalization to housekeeping gene (GAPDH) and then to untreated cells (dashed line). (*D*) Effect on MMP3 production. (*E*) Effect on IL-6, IL-8, and CCL20 production. All results are mean ± SD, *n* = 4 control and frozen shoulder fibroblasts, * indicates significant difference from untreated cells, *P < 0.05, **P < 0.01, ***P < 0.001. ^#^*P* < 0.05, ^##^*P* < 0.01, ^###^*P* < 0.001. All statistical analyses use ANOVA with Bonferroni or Dunn’s correction for multiple comparisons depending on normality.

As the TRAF6-dependent pathway in IL-17A signaling is NF-κB dependent (47, 48), we sought to elucidate whether IL-17A signaling in frozen shoulder was indeed NF-κB dependent and whether the profibrotic and inflammatory response could be blocked by utilizing a small molecule inhibitor we have previously shown to be effective in a model of tendon disease (49). The pretreatment of frozen shoulder fibroblasts with 50 nM IKKβ inhibitor, prior to cytokine exposure, was able to significantly negate the effects of IL-17A on fibroblast viability and the associated changes in cytochrome C and *BCL2* messenger RNA (mRNA) expression compared to cells pretreated with a vehicle control (Fig. 4 *A* and *B*). Furthermore, this small molecule inhibitor was also able to prevent the IL-17A–induced effects on matrix and inflammatory proteins (Fig. 4 *C*–*E*). Hence, the effects of IL-17A on frozen shoulder fibroblast appear to be mediated via NF-κB.

### Translatable Anti–IL-17A Therapy in Fibrosis.

**Fig. 5.**
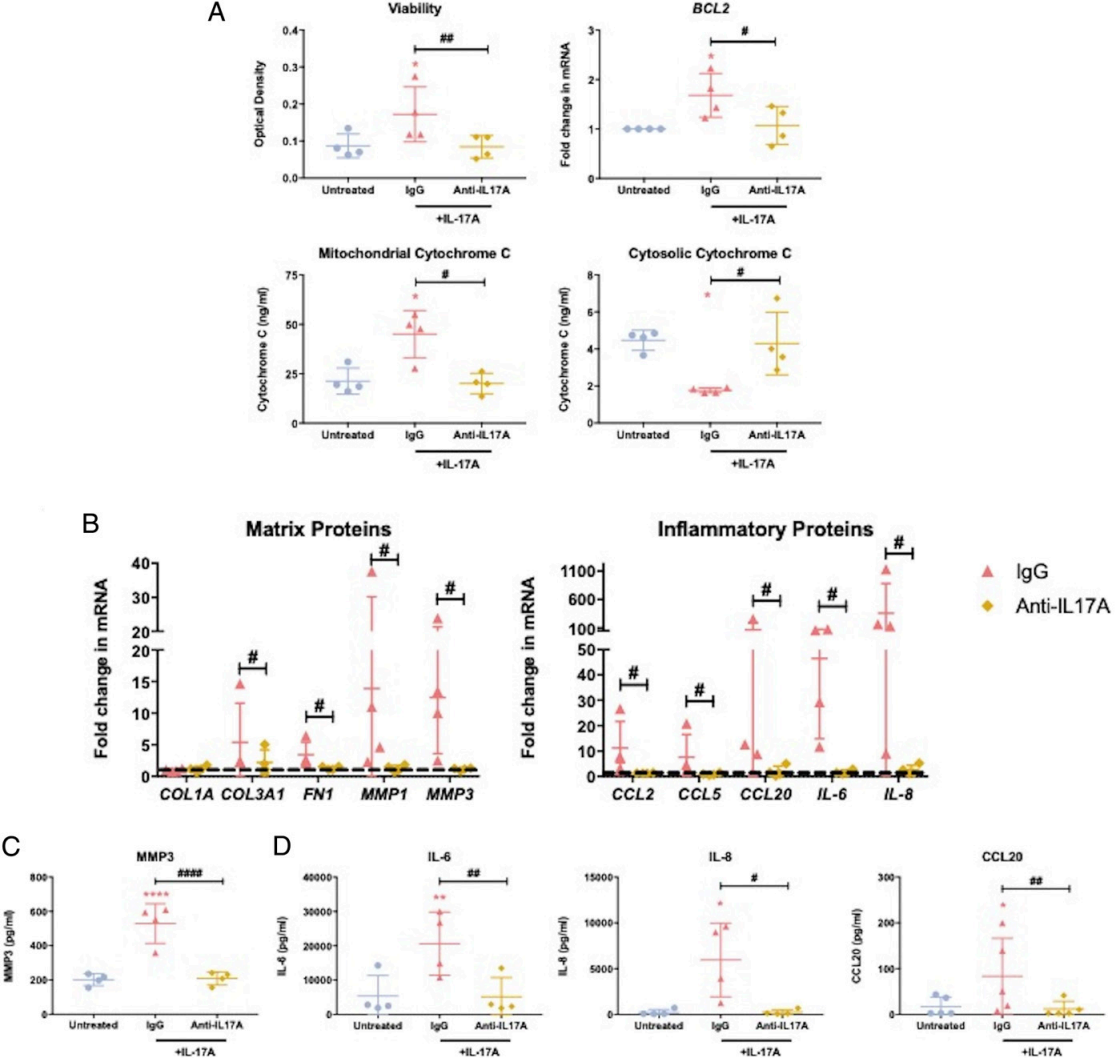
Anti–IL-17A monoclonal targeting of IL-17A induced fibrotic and inflammatory effects in frozen shoulder. Frozen shoulder fibroblasts were pretreated with an anti–IL-17A monoclonal antibody or IgG control before exposure to IL-17A (100 ng/mL). (*A*) Fibroblast’s viability, *BCL2* gene expression, and mitochondrial and cytosolic cytochrome C content. (*B*) Gene expression of matrix and inflammatory regulators. mRNA gene expression expressed as fold change following normalization to housekeeping gene (GAPDH) and then to untreated cells (dashed line). (*C*) Effect on MMP3 production. (*D*) Effect on IL-6, IL-8 and CCL20 production. All results are mean ± SD, *n* = 4 control and frozen shoulder fibroblasts, * indicates significant difference from untreated cells, *P < 0.05, **P < 0.01, ****P < 0.001, ^#^*P* < 0.05, ^##^*P* < 0.01, ^####^*P* < 0.0001. All statistical analyses use ANOVA with Bonferroni or Dunn’s correction for multiple comparisons depending on normality.

The use of an anti–IL-17A monoclonal antibody has been successful in the treatment of a number of IL-17A–driven inflammatory pathologies (28, 29). Given our data demonstrated a clear role for IL-17A in driving disease, we explored the ability of this readily available therapeutic agent to inhibit the profibrotic and inflammatory effects in frozen shoulder. Firstly, we assessed whether this monoclonal antibody could inhibit IL-17A–induced effects on fibroblast viability, *BCL2* expression, and cytochrome C content. We observed that frozen shoulder fibroblast viability was significantly lower following pretreatment with the anti–IL-17A antibody prior to IL-17A exposure compared to cells that were pretreated with IgG control (Fig. 5*A*). Additionally, the data demonstrated *BCL2* expression and mitochondrial cytochrome C content in cytokine-stimulated fibroblasts was significantly lower following anti–IL-17A pretreatment (Fig. 5*A*). Additionally, the cytosolic cytochrome C measured in anti–IL-17A–pretreated cells was significantly greater than that measured in IgG-pretreated cells (Fig. 5*A*). Furthermore, we ascertained that anti–IL-17A antibody treatment could successfully inhibit the changes in matrix and inflammatory proteins following IL-17A stimulation in diseased fibroblasts. The data indicate the anti–IL-17A monoclonal antibody could significantly inhibit the IL-17A–stimulated increase in matrix and inflammatory gene expression observed in frozen shoulder fibroblasts (Fig. 5*B*). In addition, we were able to show that the blockade of IL-17A signaling via anti–IL-17A treatment was able to significantly inhibit increased MMP3, IL-6, IL-8, and CCL20 secretion from frozen shoulder fibroblasts following IL-17A exposure (Fig. 5 *C* and *D*). Together, these data suggest IL-17A signaling inhibition may be a viable intervention of the fibrotic and inflammatory features observed in frozen shoulder.

## Discussion

Our data show the presence of IL-17A–producing T cells in frozen shoulder capable of inducing profibrotic and inflammatory effects in diseased fibroblasts through enhanced expression of the signaling receptor IL-17RA driven by a TRAF6/NF-κB–dependent pathway. Furthermore, inhibition of IL-17A signaling via IKKβ inhibition or anti–IL-17A antibodies was able to attenuate these changes seen in diseased cells. Thus, this study demonstrates that targeting cytokine-driven immune-mediated pathways in the common fibrotic condition frozen shoulder is a potential translational therapy.

Frozen shoulder has historically been described as the inflammatory fibrosis of the shoulder capsule, resulting in debilitating pain and loss of movement (13, 50). Previous investigations identified the infiltration of immune cells within the capsule in biopsies taken from patients undergoing surgery for frozen shoulder, confirming the presence of B cells, macrophages, mast cells, and T cells (9). Although other studies have also demonstrated positive macrophage staining in similar tissue biopsies (16, 51), neither have directly compared them to immune cells in the nondiseased shoulder capsule. The current study demonstrates the shift from the predominance of macrophages to DCs and T cells in the immune cell compartment within the shoulder capsule following the onset of disease. The presence of multiple DC and T cell populations in diseased capsule tissue highlights a potential critical role for these cells in the pathogenesis of frozen shoulder. While we have focused mainly on IL-17A–producing T cells, it is likely to be the case that immune cell–driven fibrosis is multidimensional, with critical roles for a number of these cell subpopulations; this should be explored further to elucidate the pathogenesis of fibrotic disorders and possible subsequent therapeutic targeting. Interestingly, the main macrophage population we found in control capsule is phenotypically similar (*LYVE1* and *MERTK* expressing [*SI Appendix*, Fig. S3*B*]) to those found in rheumatoid arthritis patients under remission that were shown to be negative regulators of inflammation and induced repair responses in synovial fibroblasts in vitro (52). We postulate this reduced presence of resolutory macrophages may indicate a loss of regulation or aberrant tissue repair, resulting in the myeloid component becoming dominated by multiple DC populations and subsequent influx of pathogenic T cells.

The presence of T cells within fibrotic pathologies is well established; in particular, IL-4– and IL-13–secreting T cells are known to induce fibrosis (6–8). However, recently there has been accumulating data demonstrating the role of IFN-γ– (53) or IL-17A– (24, 37, 54) secreting T cells in the pathogenesis of fibrotic disorders (17, 18) as well as determining pathways of repair following tissue damage (55). Similar to these data, we demonstrate that IL-17A can directly promote a number of profibrotic mechanisms, including fibroblast survival and enhanced gene expression of proteins associated with matrix deposition. These profibrotic characteristics have previously been attributed to the elevated levels of the cytokine TGF-β in frozen shoulder (12, 14, 56). However, our data suggest the presence of IL-17A can also encourage the fibrotic hallmarks of frozen shoulder.

**Fig. 6.**
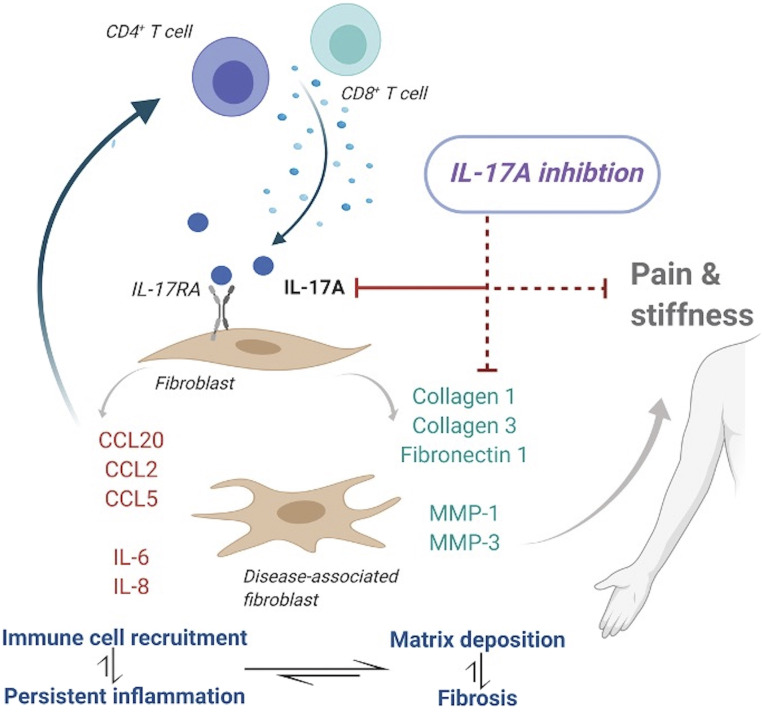
Targeting IL-17A in frozen shoulder. Schematic diagram illustrating possible IL-17A–driven pathogenesis in frozen shoulder. The presence of IL-17A–secreting T cells results in the fibrosis, inflammation, and further recruitment of pathogenic T cells, resulting in disease chronicity, which can be attenuated by use of an anti–IL-17A monoclonal antibody.

The inflammatory effect effects of IL-17A on fibroblasts are well established (17, 25, 37, 47). We were able to support these previous findings by demonstrating increased expression of a number of cytokines and chemokines from frozen shoulder fibroblasts in response to IL-17A stimulation, which would result in a conducive inflammatory environment for the recruitment and activation of T cells. Of particular note was the ability of IL-17A to initiate a significant release of CCL20 from frozen shoulder fibroblasts. One of the main functions of this chemokine is the recruitment of CCR6^+^ IL-17A–producing T cells in disease (45, 57). We postulate that there may be a positive chemotactic feedback loop which results in the perpetual recruitment of IL-17A–producing cells due to continuous IL-17A exposure and subsequent CCL20 release (Fig. 6).

IL-17A can signal through both NF-κB–dependent and independent mechanisms; however, the current data indicate IL-17A signaling in these cells was NF-κB dependent. The NF-κB signaling pathway plays a central role in inflammation and cell survival (58). By selectively inhibiting this pathway, we were able to successfully impede the profibrotic and inflammatory IL-17A–induced changes in frozen shoulder fibroblasts. We have previously demonstrated the use of a small molecule inhibitor of NF-κB can be of therapeutic benefit to another connective tissue disease (49), and these current data further support the potential of small molecule inhibitors as prospective therapeutic agents.

The downstream effects of NF-κB and IL-17A are ultimately governed by the interaction of IL-17A with its cognate receptor(s). In the case of frozen shoulder, IL-17RA appears to be the principal signaling receptor. The lack of IL-17RA has been shown to protect mice from repeated challenges that cause liver injury, profibrotic cytokine expression, and fibrosis with collagen deposition (18). Although we have previously shown that frozen shoulder fibroblasts produce more inflammatory proteins compared to nondiseased cells (15), here we also demonstrate the magnitude of IL-17A downstream effects are greater in diseased cells compared to normal as a result of increased expression of IL-17RA in diseased cells. Interestingly, this appears to be a feature of diseased fibroblasts from a number of pathologies (17, 23), indicating that either mechanical or inflammatory trauma can render these cells more susceptible to IL-17A, resulting in disease chronicity. Thus, the targeting of this cytokine–receptor interaction is of therapeutic potential in a number of pathologies. Furthermore, the role of IL-17A is well documented in a number of inflammatory diseases such as psoriatic arthritis, ankylosing spondylitis, and rheumatoid arthritis (35, 36). Psoriatic arthritis and ankylosing spondylitis in particular have been found to be clinically responsive to anti–IL-17A treatment (28, 29). This has recently been extended to musculoskeletal soft tissue tendon disease with the completion of a Phase II trial (30). Furthermore, there is increasing evidence to suggest that IL-17A may also have a central role in the pathogenesis of systemic sclerosis (59, 60), a progressive fibrotic disorder. These studies, in addition to current data, indicate that fibrosis and inflammation are not two distinct means of disease development; rather, these processes are intertwined in chronic diseases. Thus, the data from the present study further support the potential benefit of exploring anti–IL-17A as a nonsurgical intervention in a debilitating chronic fibrotic disorder in which pharmaceutical treatments are limited.

We acknowledge that our experimental datasets lack in vivo animal data. Although a number of animal models have been used to study cytokine-driven fibrotic pathogenesis, there is no well-described in vivo model available for frozen shoulder. Those that have described in vivo models have tended to inject TGF-β (56) into the joint or use joint immobilization (61) to directly explore the fibrotic pathways more so than the inflammatory mechanisms of disease, which may result in fibrosis. Additionally, in the current study, we primarily measure gene expression of matrix proteins rather than the physical proteins following IL-17A exposure. However, this was principally to highlight changes in these matrix protein genes that were due directly to IL-17A stimulation, and any subsequent intervention in the IL-17A pathway would impede changes in these genes. Furthermore, we have previously demonstrated that diseased fibroblasts from frozen shoulder have an “activated” phenotype which accompanies elevated baseline inflammation compared to control fibroblasts (15). This inflammatory phenotype could influence some of the in vitro effects observed in the current study; however, by directly inhibiting the signaling and cytokine–receptor binding, we believe we have demonstrated the clear and direct effect of IL-17A on diseased fibroblasts. Lastly, we note that our control capsule samples were younger than the frozen shoulder samples, and thus, some aspects may be due to aging effects. However, obtaining nondiseased control tissue from human subjects is extremely difficult, particularly from older patients who predominantly have some degree of deterioration of soft tissue irrespective of diagnosis. We have consciously used capsule tissue as a control rather than use healthy soft tissue obtained from another anatomical site as a pseudocontrol.

In summary, the current study establishes a role for IL-17A–driven pathogenesis in frozen shoulder. The shift from a primarily macrophage-dominant to a T cell–rich immune repertoire signifies the importance of the immune cell landscape in regulating disease chronicity in frozen shoulder. In addition, through dissection of the signaling pathways involved in the profibrotic and inflammatory processes, we suggest that targeting of IL-17A signaling with readily available pharmaceutical agents may provide novel nonsurgical early interventions for frozen shoulder.

## Materials and Methods

### Study Design.

The purpose of the current study was to dissect the role of inflammation, particularly the IL-17A pathway, in the manifestation of frozen shoulder. Additionally, we aimed to identify if this pathway could be suitably targeted for therapeutic intervention. We aimed to identify changes in immune cell landscape influencing the diseased environment in frozen shoulder, particularly IL-17A–secreting cells. Next, we investigated the fibrotic and inflammatory effects of IL-17A, in vitro, and then sought to investigate the mechanism by which diseased cells were more responsive to IL-17A stimulation. Finally, we sought to identify if the pathways identified could be targeted using readily available pharmacological agents that could inhibit the pathways involved in frozen shoulder.

### Study Approval.

Human study procedures and protocols were approved by the National Health Service West of Scotland Ethics Committee (Research Ethics Committee 11/S0704/7). Full informed consent was obtained from all patients. Sample size for tissue- and cell-based assays were determined based on sample availability and technical needs.

### Clinical Samples.

Frozen shoulder tissue was collected from patients with primary frozen shoulder undergoing surgical arthroscopic capsular release (*n* = 10, 7 females, 3 males, mean age 52 ± 10.2 SEM). Control tissue was obtained from patients undergoing arthroscopic stabilization surgery, unaffected by any primary shoulder disease (*n* = 10, 3 females, 7 males, mean age 31.3 ± 14.6 SEM). Tissue was characterized as macroscopically at the time of surgery and microscopically by hematoxylin and eosin staining prior to any control experiments (*SI Appendix*, Fig. S1).

### Histology.

Tissue was fixed in 4% paraformaldehyde and embedded in paraffin using standard techniques. Sections (5 μm) were obtained and stained with hematoxylin and eosin (Vector Laboratories).

### Ex Vivo Stimulation.

Tissue was digested in Liberase TM (Sigma-Aldrich) in Roswell Park Memorial Institute (RPMI) media for a maximum of 2 h at 37 °C. Digested tissue was then passed through a 100-μm cell strainer, pelleted by centrifugation at 350 *g* for 5 min, and the supernatant was discarded. This was repeated twice. Cells were either harvested for flow cytometry or underwent the appropriate stimulation for 24 listed prior to harvesting for intracellular flow cytometry. T cells were activated by stimulating the disaggregated cell suspension for 24 h with Phorbol 12-myristate 13-acetate (50 ng/mL) plus ionomycin (1 μg/mL) (all Sigma-Aldrich).

### Fibroblast Isolation and Culture.

Control and diseased fibroblasts were extracted from stabilization and frozen shoulder capsule tissue, respectively. Tissue was disaggregated as described above and cell suspension resuspended in supplemented culture medium (RPMI with 10% fetal bovine serum 1% penicillin/streptomycin and 1% L-glutamine [all Invitrogen]) at 37 °C, 5% CO_2_, and 95% humidity. Cultures were maintained at 37 °C in a humidified atmosphere of 5% CO_2_. Cells were subcultured and trypsinized at sub confluency and used at passage 2 to 4. Fibroblasts used for in vitro experiments were analyzed via qPCR to assess any endothelial or immune cell contamination (*SI Appendix*, Table 1).

### In Vitro Fibroblast Stimulation.

Cells were plated in 24-well culture plates at a density of 2.5 × 10^4^ cells per well with 0.5 mL supplemented culture medium and allowed to adhere 48 h prior to stimulation. Cells were stimulated with recombinant human IL-17A (1 to 100 ng/mL) (BioLegend) diluted in supplemented culture medium for 24 h unless stated below. After the stimulation period, supernatants were collected and cells harvested for the appropriate assay.

For cell proliferation and viability assays and cytosol/mitochondrial fractionation, cells were serum starved overnight prior to stimulation; the stimulation period was 72 h.

In the inhibitor studies, cells were seeded as above for 48 h. The cells were then pretreated with the appropriate inhibitor or control for 30 min prior to IL-17A stimulation for 24 h. The concentrations and relevant control are as follows: 50 mM IKKβ inhibitor VIII (Merck) and dimethyl sulfoxide control, 10nM anti–IL-17A (Secukinumab, Novartis), and IgGκ (BioLegend) compared to vehicle-treated controls. Following the stimulation period, supernatants were collected and cells harvested for the appropriate assay. The supernatant was sampled to determine inflammatory cytokine concentration, and RNA was isolated from cell lysates.

### Viability Assay.

Cell viability was assessed using the TOX1kit (MTT 3-(4,5-dimethylthiazol-2-yl)-2,5-diphenyltetrazolium bromide based) as per the manufacturers protocol (Sigma-Aldrich).

### BrdU Assay.

Cell proliferation was assessed using the BrdU Cell Proliferation Kits per the manufacturers protocol (Sigma-Aldrich). BrDU reagent was added in the final 24 h of the experiment.

### Mitochondria/Cytosol Fractionation.

Enriched mitochondrial and cytosolic fractions were obtained from fibroblasts using a Mitochondria/Cytosol Fractionation Kit (Abcam) as per the manufacturers protocol.

### Enzyme-Linked Immunosorbent Assay.

Chemokine, cytokine, cytochrome C, and MMP3 concentration was determined using enzyme-linked immunosorbent assay kits as per the manufacturers protocol; CCL20 (BioLegend), cytochrome C, IL-6, IL-8 (all Invitrogen), and MMP3 (R&D Systems).

### Western Blot.

Cells were seeded in 6-well plates as described above for 48 h but at a density of 1 × 10^6^ in 3 mL culture medium. Following this period, the culture medium was replaced with 2 mL fresh culture medium for 2 h. Cells were lysed with radioimmunoprecipitation assay lysis buffer supplemented with Halts protease and phosphatase inhibitors (Thermo Fisher Scientific). Total protein was quantified with the Pierce BCA Protein Assay Kit (Thermo Fisher Scientific). Equal amounts of total cell lysate (30 mg per well) were subjected to sodium dodecyl sulfate electrophoresis on 4 to 12% Bis-Tris gels (Thermo Fisher Scientific). Gels were run at a constant voltage of 100 mV (PowerPac 3000, Bio-Rad) and transferred onto a nitrocellulose membrane with the iBlot2 Transfer System (Thermo Fisher Scientific) as per manufacturer’s instructions. Immunoblotting was completed for GAPDH, IL-17RA, TRAF6 (all Cell Signaling Technology), and IL-17RC (Abcam). A Li-Cor Azure C500 imager was used to visualize immunoblots, and ImageJ software (NIH) was used for quantification.

### Flow Cytometry.

Single-cell suspensions were labeled with fluorophore-conjugated anti-mouse antibodies (Table 1) and Zombie viability dye at recommended dilutions following the manufacturers’ recommendations (BioLegend). Data were acquired on a BD LSR II using FACS DIVA software with automated compensation (BD Biosciences). Compensation data were acquired using single stained BD Comp beads as per manufacturer’s instructions (BD Biosciences). All data were analyzed using FlowJo software (BD Biosciences).

**Table 1.** Flow cytometry antibodies used in current study

Antibody	Cells identified
CD45	Immune cells
CD3	T cells
CD4	CD4 T cells
CD8	CD8 T cells
CD19	B cells
CD64	Myeloid cells
CD117	Mast cells
IFN-γ	Interferon-γ producing
IL-17A	IL-17A producing
Fixable viability dye	All viable cells

### Single-Cell RNA Sequencing.

Single-cell suspensions of cells were derived from freshly digested tendon biopsies following surgical excision as described above. Live cells were sorted using a FACS ARIA III. Isolated cells were lysed, and then RNA was reverse transcribed and converted to complementary DNA (cDNA) libraries for RNA sequencing analysis using a Chromium Controller and Chromium Single-Cell 3′ v2 Reagent kit (10x Genomics) following the manufacturer’s protocol. Pooled libraries were used for sequencing on a HiSeq 4000 (Illumina) to a depth of ∼30,000 reads per cell. The alignment of reads to the genome and generation of gene counts per cell were performed by Cell Ranger software (10x Genomics). Quality control was performed on each sample, and poor-quality cells were removed on the basis of number of genes expressed (>200), of unique molecular identifiers, and percentage of mitochondrial reads mapped (>5%). Using the Seurat Package (Sajita Lab), we normalized and scaled the data. Following this, principal components analysis and high-quality cells were clustered using a graph-based routine implemented in the Seurat R package (Satija Lab).

### Gene Expression.

Cells were harvested in PureLink lysis buffer containing 1% 2-mercaptoethanol, and RNA was extracted using mini columns according to the PureLink protocol (Thermo Fisher Scientific). RNA concentration and purity were determined using a spectrophotometer (Nanodrop 2000, Thermo Scientific), and 100 ng RNA was converted to cDNA using the High-Capacity cDNA Reverse Transcription Kit (Thermo Fisher Scientific) according to manufacturer’s instructions. cDNA was diluted 1 in 5 using RNase free water. qPCR was performed using PowerUp Sybr Green Mastermix (Thermo Fisher Scientific). Each sample was run in duplicate and normalized to endogenous control (GAPDH) following confirmation that there was no more than 0.25 cycles difference in the control gene between each treated condition. Data represents relative mRNA expression to housekeeping gene (2^−ΔCT^) or fold change from untreated cells (2^−ΔΔCT^).

Primers (Integrated DNA Technologies) were as follows:

*GAPDH* (f) 5′-TCG​ACA​GTC​AGC​CGC​ATC​TTC​TTT-3′, (r) 5′-ACC​AAA​TCC​GTT​GAC​TCC​GA CCTT-3′, *PDPN* (f) 5′-CTT​GAC​AAC​TCT​GGT​GGA-3′, (r) 5′-GGG​CTT​GGA​CTT​GTT​CTT​G-3′, *CD45* (f) 5′-AAA​GCC​CAA​CAC​CTT​CCC-3′, (r) 5′-AGT​AGC​TAT​TGT​TGT​GGT​TGA​AAT​G-3′, *CD31* (f) 5′-ATT​CCT​GAA​GTC​CGG​ATC​TAT​G-3′, (r) 5′-GGA​CTG​GGC​ACT​CCT​TC-3′, *COL1A1* (f) 5′-CAA TGC TGC CCT TTC TGC TCC-3′, (r) 5′-CAC TTG GGT GTT TGA GCA TTG-3′, *COL3A1* (f) 5′-TAT CGA ACA CGC AAG GCT GTG-3′, (r) 5′-GGC CAA CGT CCA CAC CAA ATT-3′, *TNC* (f) 5′-CTT​TGG​CTG​GGT​TGC​TTG​AC-3′ (r) 5′-GTG​CCA​GGA​GAC​CGT​ACC​AC-3′, *FN1* (f) 5′-TTG TAC TTG CCT GGG AGA AG-3′, (r) 5′-CTC​CAG​GTG​TCT​CCA​ATT​CTA​T-3′, *MMP1* (f) 5′-TGC GCA CAA ATC CCT TCT-3′, (r) 5′-AGC CCA GTA CTT ATT CCC TTT G-3′, *MMP3* (f) 5′-ACC CGA CCT TAC ATA CAG GAT T-3′, (r) 5′-GTC ACC TCT TCC CAG ACT TTC-3′, *α-SMA* (f) 5′-CCT CCC TTG AGA AGA GTT ACG A-3′, (r) 5′-GAC TCC ATC CCG ATG AAG GAT-3′, *BCL2* (f) 5′-AGG CTG GGA TGC CTT TGT -3′, (r) 5′-GAC TTC ACT TGT GGC CCA GAT A-3′, *BAX* (f) 5′-GGA GCT GCA GAG GAT GAT TG -3′, (r) 5′-AGT TGA AGT TGC CGT CAG AA-3′, *CCND1* (f) 5′-TGT GCC ACA GAT GTG AAG TT-3′, (r) 5′-GTA GTA GGA CAG GAA GTT GTT GG-3′, *IL-6* (f) 5′-CAC TCA CCT CTT CAG AAC GAA T-3′, (r) 5′-GCT GCT TTC ACA CAT GTT ACT C-3′, *IL-8* (f) 5′-GTG CAT AAA GAC ATA CTC CAA ACC-3′, (r) 5′-GCT TTA CAA TAA TTT CTG TGT TGG C-3′, *IL-17A* (f) 5′-TCT GCT ATT CTG GAT ACT GCT TTC -3′, (r) 5′-TCT GTG GAG TAC TTT GTT CAC C -3′, *IL-17B* (f) 5′-TGT CAC GGA TGA AAC CGT ATG-3′, (r) 5′-TTC TCT GGG CCA GCT CT-3′, *IL-17C* (f) 5′-CCA CCA TGA CGC TCC TC-3′, (r) 5′-TCA GCC GAG TAG CAG TGT-3′, *IL-17D* (f) 5′-TGG GCC TAC AGA ATC TCC TA-3′, (r) 5′-GAC GGT GGG CAT GTA GAC-3′, *IL-17E* (f) 5′-CCC TGG AGA TAT GAG GCT T-3′, (r) 5′-TGC ACT GAC CTG GTA CAT-3′, IL-17RA (f) 5′-GAT TCA CCC TCG AAA CCT GA-3′, (r) 5′-ATG CTG GCG TCT GTC TG-3′, IL-17RC (f) 5′-CGT GCA TCT GGT TCT GAA TG-3′, (r) 5′-GCG GTC CAG TCA GGT TT-3′, *CCL2* (f) 5′-CTC AGC CAG ATG CAA TCA ATG-3′, (r) 5′-TGC TGC TGG TGA TTC TTC TAT-3′, *CCL5* (f) 5′-GCT GCT TTG CCT ACA TTG C-3′, (r) 5′-CTT TCG GGT GAC AAA GAC GA-3′, *CCL20* (f) 5′-GTC TTG GAT ACA CAG ACC GTA TT-3′, (r) 5′-GTG TGA AAG ATG ATA GCA TTG ATG T-3′.

### Statistical Analysis.

All data are shown as means ± SD. All statistical analyses were performed following a Shapiro–Wilk normality test on the data using GraphPad Prism 7 software. Individual tests for each dataset are described in the relevant figure legend. A *P* value < 0.05 was considered significant.

## Supplementary Material

Supplementary File

## Data Availability

Raw UMI counts and normalized expression values for single-cell RNA-Seq are publicly available at https://owncloud.gla.ac.uk/cloud/s/e4pZdcSzN1EClhE. Individual sequencing read data will be available on request under the condition of approval of the ethics committee of Glasgow University and material transfer agreement. All other study data are included in the article and/or *SI Appendix*.
