# Supplementary material for: Translational targeting of inflammation and fibrosis in frozen shoulder: Molecular dissection of the T cell/IL-17A axis
Source: Proc Natl Acad Sci U S A. 2021 Sep 20;118(39):e2102715118. doi: 10.1073/pnas.2102715118 (PMC8488623; doi:10.1073/pnas.2102715118)
Supplement: Supplementary File [file pnas.2102715118.sapp.pdf]

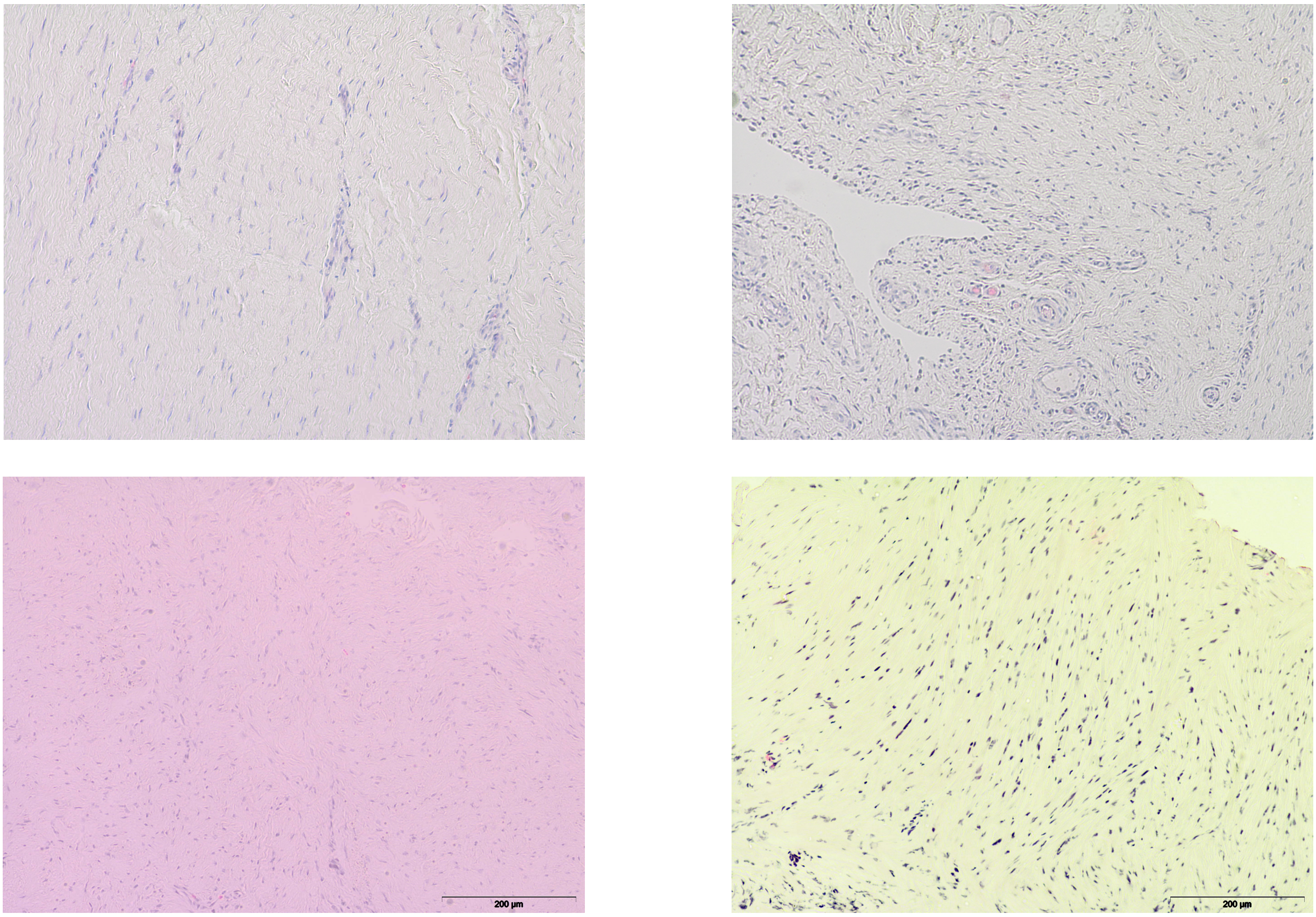

Control

Frozen Shoulder

Supplementary 1

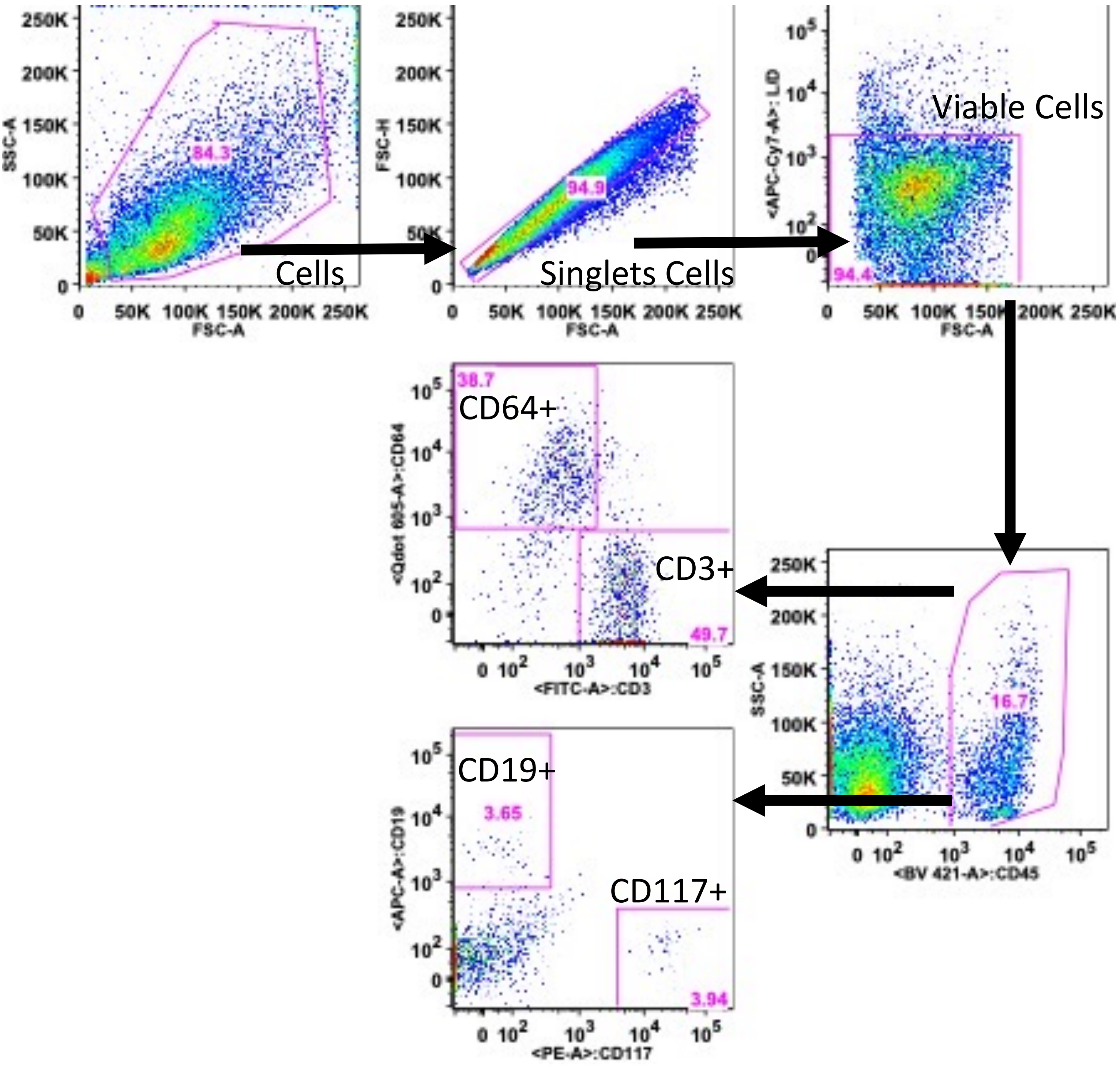

Supplementary 2

a

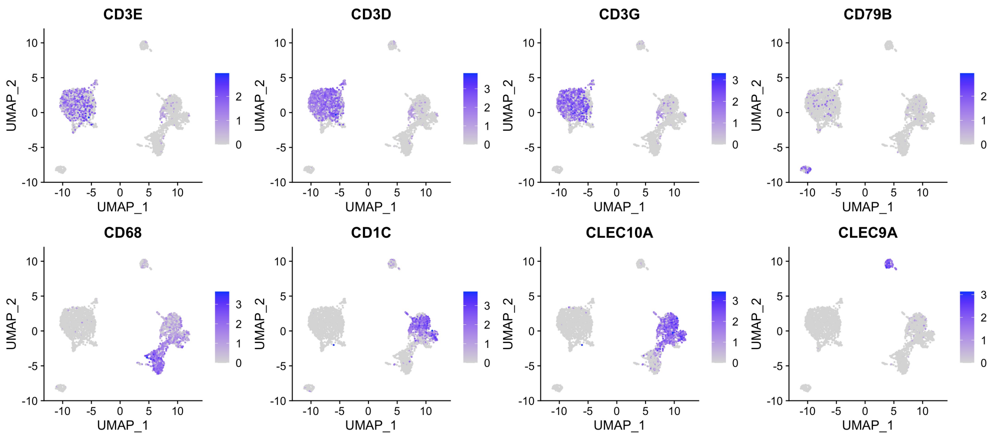

b

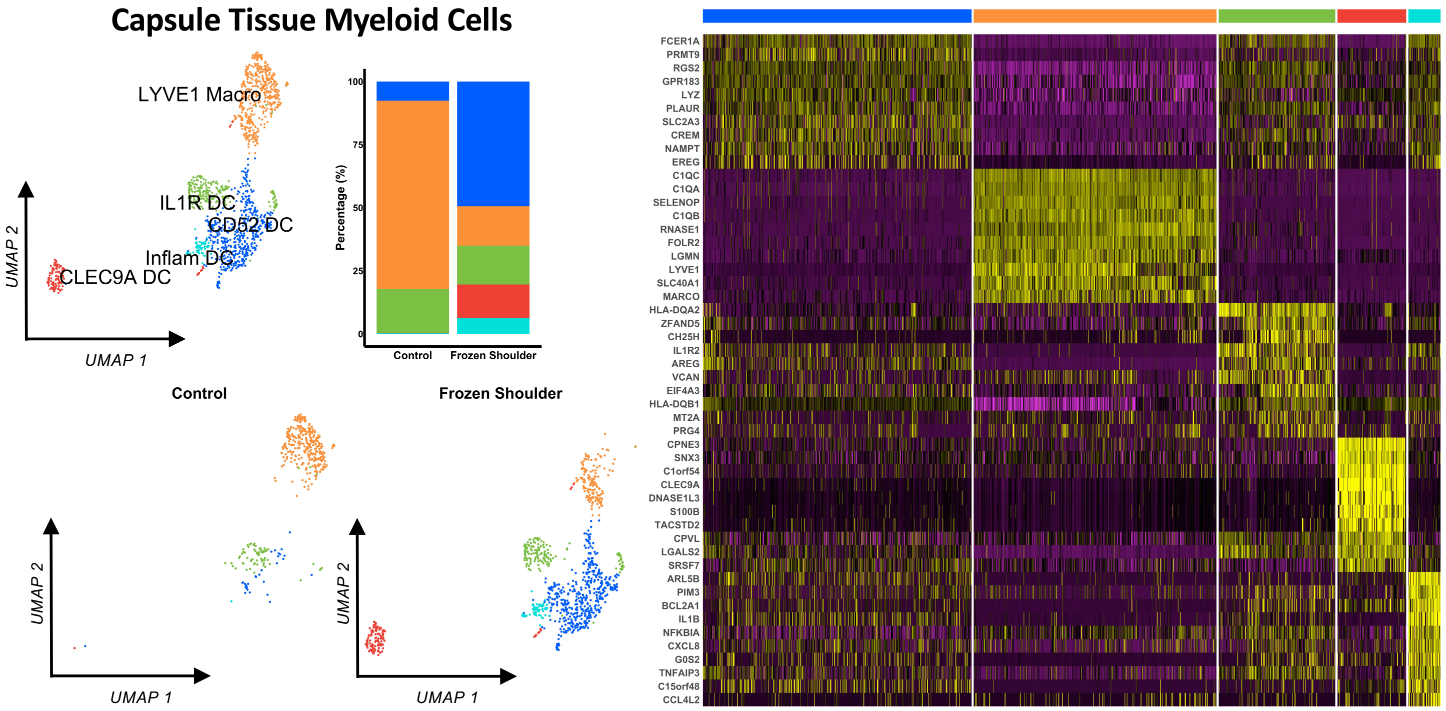

c

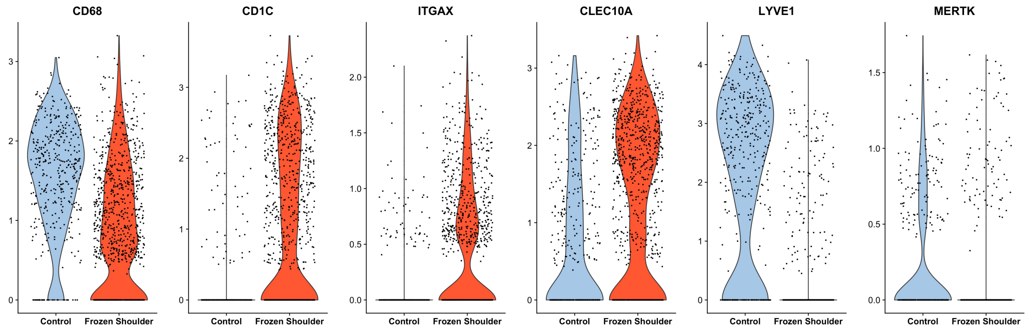

Supplementary 3

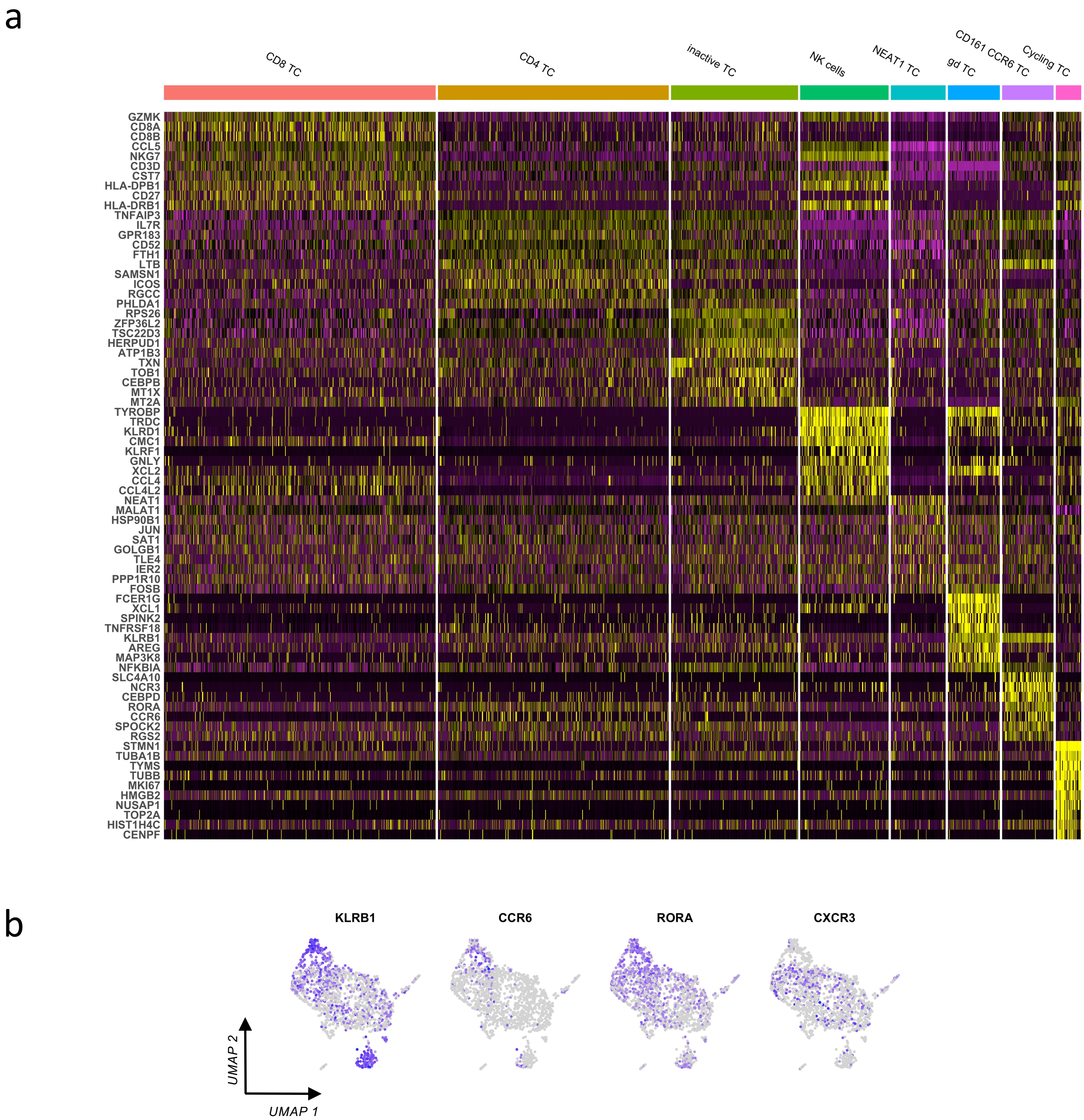

Supplementary 4

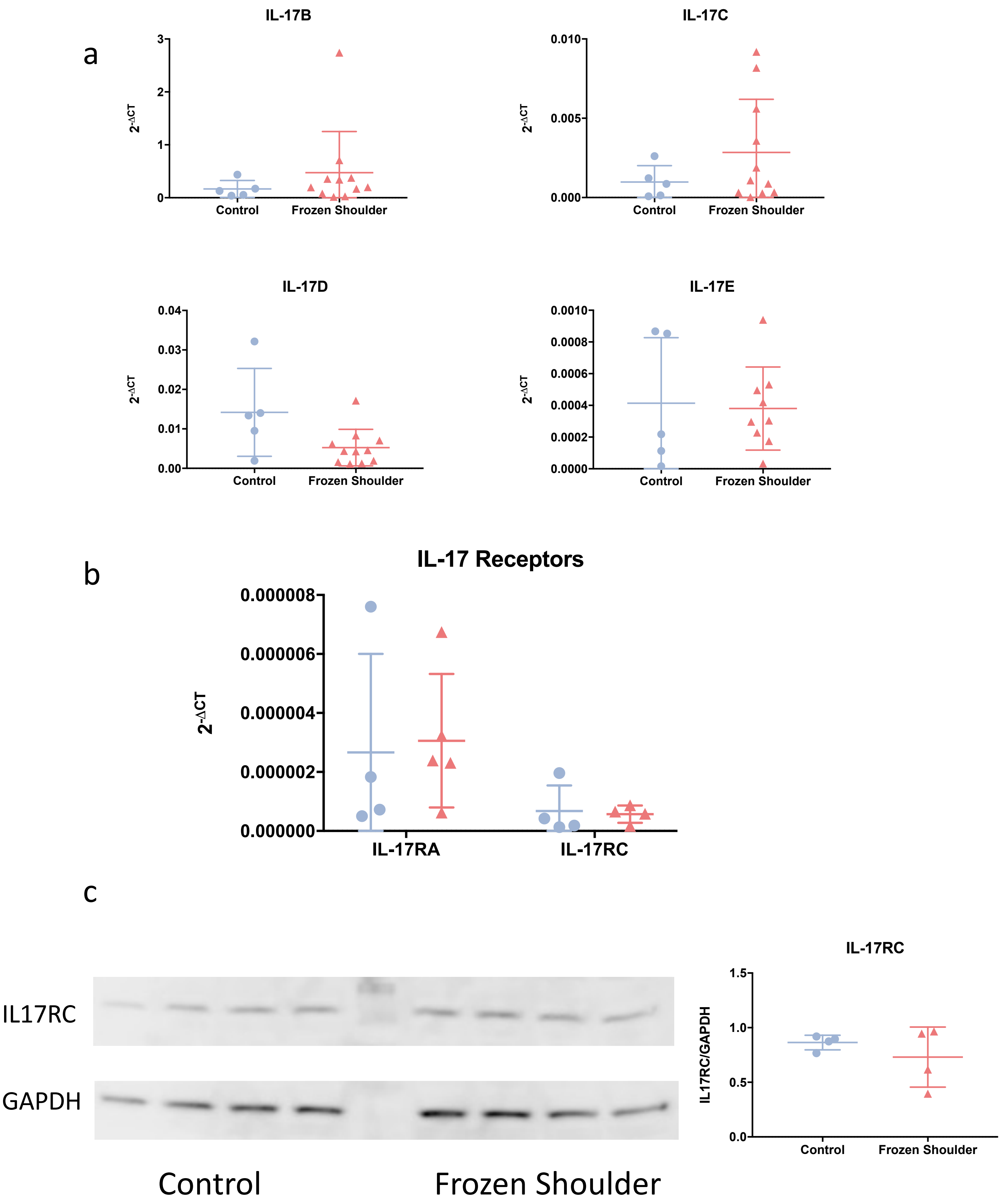

Supplementary 5

a

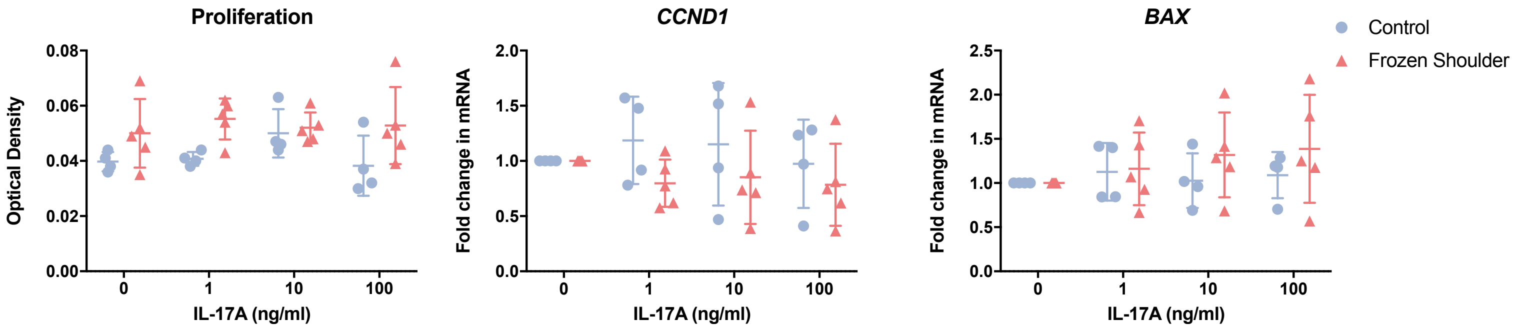

b

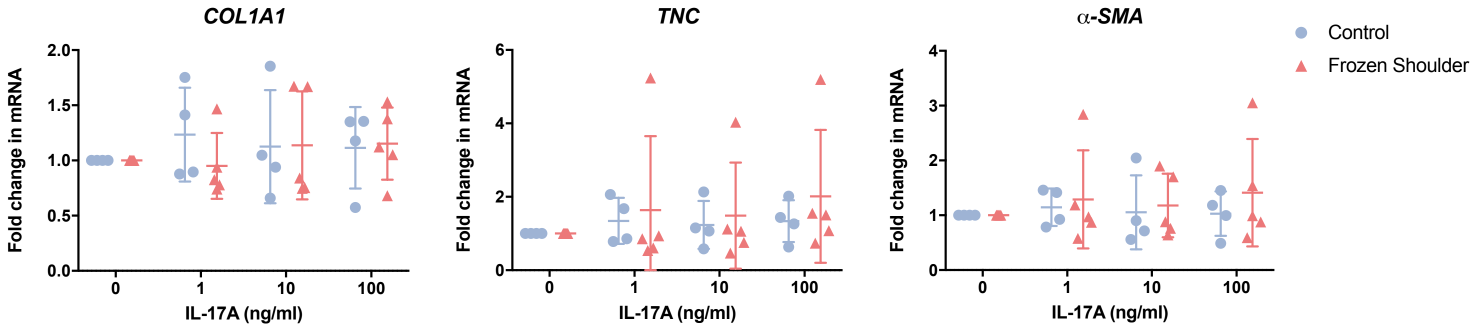

c

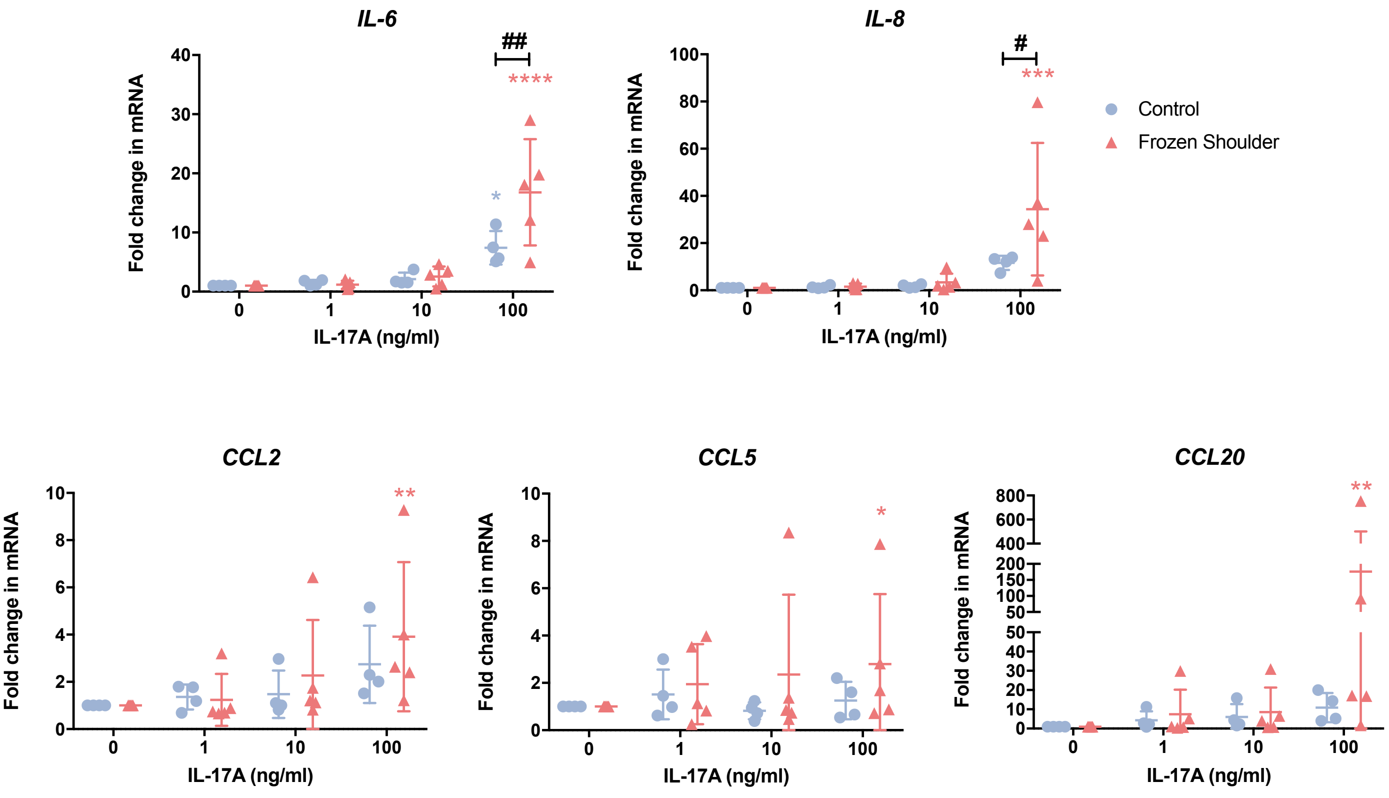

Supplementary 6

Supplementary Table

**Cells**	***PDPN***	***CD31***	***CD45***
Control	26.57±0.50	34.13±1.36	34.61±0.66
Frozen Shoulder	26.42±1.09	34.55±1.09	35.73±0.51
Macrophage	31.21	29.04	24.65
HUVEC	n.d.	21.17	34.85

Supplementary Table 1. Endothelial, immune and fibroblast marker gene expression of cultured fibroblast. CT values of *PDPN* (fibroblast), *CD31*(endothelial) and *CD45* (immune) in cultured fibroblasts used for experiments. Results are mean ± SD, n=4 control capsule and n=5 frozen shoulder, with HUVEC and macrophage cDNA used as a positive control for endothelial and immune cells, respectively.

Supplementary Figure 1. Histology of control and frozen shoulder capsule. (A) Representative H&E images of 2 control and 2 frozen shoulder capsule tissues, 10x magnification.

Supplementary Figure 2. Identification of immune cells in shoulder capsule. Representative FACS plots from disaggregated frozen shoulder tissue demonstrating gating strategy to identify immune cell populations.

Supplementary Figure 3. Inflammatory cells in shoulder capsule. (A) UMAP embedding of single-cell RNA-seq of immune cells with cell marker gene expression in scaled(log(UMI+1)) in shoulder capsule (n=7, k=3347). (B) UMAP embedding and distribution of five delineated myeloid cell populations (LYVE1 1 Macrophages, IL-1 receptor DCs, CD52 DCs, inflammatory DCs, CLEC9A DCs) in all shoulder capsule tissue (n=7, k=1357) and split into shoulder capsule of control (n=3, k=408) and frozen shoulder tissue (n=4, k=949). Heatmap of marker genes of myeloid cell populations in all shoulder capsule tissue single-cell RNA-seq data, displaying average row scaled expression in log(UMI + 1). (C)Violin plots of marker genes in myeloid cells from control and frozen shoulder capsule tissue. Data from single-cell RNA-seq, displaying average row scaled expression in log(UMI + 1).

Supplementary Figure 4. T cells in shoulder capsule. (A) Heatmap of marker genes of eight T cell populations in all shoulder capsule tissue single-cell RNA-seq data, displaying average row scaled expression in log(UMI + 1). (B) UMAP embedding of single-cell RNA-seq of T Cells with cell marker gene expression in scaled(log(UMI+1)) from all shoulder capsule tissue.

Supplementary Figure 5. IL-17 isoforms and receptor expression. (A) IL-17 isoforms expression shoulder capsule. IL-17B, IL-17C, IL-17D and IL-17E expression in control and frozen shoulder capsule tissue. Results are mean ± SD, n=5 control capsule and n=10 frozen shoulder. Statistical analysis using unpaired t-test or Mann-Whitney rank-sum test depending on normality. (B) IL-17RA and IL-17RC expression in control and frozen shoulder fibroblasts. Results mean ± SD, n=4 control capsule and n=5 frozen shoulder mRNA gene expression expressed as $2^{-\Delta CT}$ following normalisation to housekeeping gene (GAPDH). Statistical analysis using unpaired t-test or Mann-Whitney rank-sum test depending on normality. (C) IL-17RC protein expression in cultured fibroblasts from control and frozen shoulder capsule. Image of western blot of protein from control and frozen shoulder fibroblasts immunoblotted for GAPDH and IL-17RC. Graph illustrates IL-17RC protein quantification relative to housekeeping (GAPDH), mean ± SD, n=4 control and frozen shoulder fibroblasts. Statistical analysis using Mann-Whitney rank-sum test depending.

Supplementary Figure 6. Effect of recombinant IL-17A on proliferation and gene expression of control fibroblast and frozen shoulder fibroblasts (A) Effect on fibroblast proliferations and *CCND1* and *BAX* gene expression. (B) *COL1A1, TNC* and *α-SMA* gene expression. (C) *IL-6, IL-8, CCL2, CCL5,* and *CCL20* gene expression. All results are mean ± SD, n=4 control fibroblasts and n=5 frozen shoulder fibroblasts. All Statistical analysis using 2-way ANOVA with Dunnets correction or Sidak's test for multiple comparisons. * Indicates significant difference from untreated cells, *p<0.05, **p<0.01, ***p<0.001, ****p<0.001. #p<0.05, ##p<0.01.
